# The Organoid Cell Atlas

**DOI:** 10.1038/s41587-020-00762-x

**Published:** 2020-12-31

**Authors:** Christoph Bock, Michael Boutros, J. Gray Camp, Laura Clarke, Hans Clevers, Juergen A. Knoblich, Prisca Liberali, Aviv Regev, Anne C. Rios, Oliver Stegle, Hendrik G. Stunnenberg, Sarah A. Teichmann, Barbara Treutlein, Robert G. J. Vries, the Human Cell Atlas ‘Biological Network’ Organoids

**Affiliations:** 1 CeMM Research Center for Molecular Medicine of the Austrian Academy of Sciences, Vienna, Austria; 2 Institute of Artificial Intelligence and Decision Support, Center for Medical Statistics, Informatics, and Intelligent Systems, Medical University of Vienna, Vienna, Austria; 3 Division Signaling and Functional Genomics, German Cancer Research Center (DKFZ), Heidelberg University, Heidelberg, Germany; 4 Institute of Molecular and Clinical Ophthalmology Basel, Basel, Switzerland; 5 European Molecular Biology Laboratory, European Bioinformatics Institute, Wellcome Genome Campus, Hinxton, UK; 6 Oncode Institute, Hubrecht Institute, Royal Netherlands Academy of Arts and Sciences, Utrecht, the Netherlands; 7 University Medical Center Utrecht, Utrecht, the Netherlands; 8 Institute of Molecular Biotechnology of the Austrian Academy of Sciences (IMBA), Vienna Biocenter (VBC), Vienna, Austria; 9 Friedrich Miescher Institute for Biomedical Research (FMI), Basel, Switzerland; 10 Klarman Cell Observatory, Broad Institute of MIT and Harvard, Cambridge, MA USA; 11 Howard Hughes Medical Institute, Koch Institute of Integrative Cancer Research, Department of Biology, Massachusetts Institute of Technology, Cambridge, MA USA; 12 Princess Máxima Center for Pediatric Oncology, Utrecht, the Netherlands; 13 Cancer Genomics Center, Utrecht, the Netherlands; 14 Divison of Computational Genomics and Systems Genetics, German Cancer Research Center (DKFZ), Heidelberg, Germany; 15 European Molecular Biology Laboratory, Genome Biology Unit, Heidelberg, Germany; 16 Wellcome Sanger Institute, Wellcome Genome Campus, Hinxton, UK; 17 Cavendish Laboratory, University of Cambridge, Cambridge, UK; 18 Eidgenössische Technische Hochschule (ETH) Zurich, Department of Biosystems Science and Engineering, Basel, Switzerland; 19 Foundation Hubrecht Organoid Technology, Utrecht, the Netherlands; 20 Human Cell Atlas Executive Office, Broad Institute of MIT and Harvard, Cambridge, MA USA; 21 Human Cell Atlas Executive Office, Wellcome Sanger Institute, Wellcome Genome Campus, Hinxton, UK

**Keywords:** Stem cells, Sequencing, Target identification, Gene regulatory networks, Cells

**To the Editor** *—* Human organoids hold tremendous potential for biomedical applications^1–3^ (Fig. 1a,b). These three-dimensional structures of cultured cells recapitulate important aspects of in vivo organ development and biological function. They provide tractable in vitro models of human physiology and pathology, thereby enabling interventional studies that are difficult or impossible to conduct in human subjects. For example, organoids allow genetic and pharmacological manipulation in a complex cellular context that reflects human biology, and they enable investigations of the early stages of organ development and disease onset. Human organoids complement (and may eventually replace) animal models in many areas of preclinical drug development. Moreover, they provide patient-specific ‘avatars’ for drug development and precision therapies, including treatments for cancer, rare genetic diseases (such as cystic fibrosis) and complex multifactorial disorders (such as epilepsy). Finally, they promise to contribute to regenerative medicine, with the goal of producing functional biological structures that can be transplanted into patients.

**Fig. 1:**
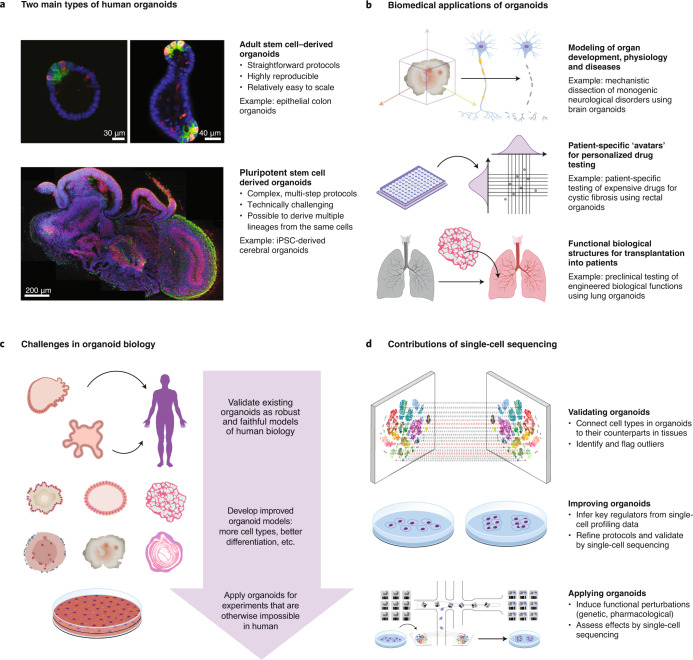
Combining organoids and single-cell technology for biomedical discovery and regenerative therapy. **a**, Organoids can be derived from adult stem cells, which are already committed to a specific cell lineage, tissue and/or organ, or from pluripotent cells, which can give rise to a broad set of cell types but requires more complex differentiation protocols. **b**, Human organoids are broadly useful for in vitro modeling of biological functions, drug testing and regenerative medicine. **c**, Key challenges for advancing organoid research include characterization and validation of organoids, development of new organoid protocols, and applications of organoids in basic biology and biomedical research. **d**, Single-cell technologies promise to advance organoid research by enabling systematic validation of the derived organoids, by informing organoid protocol development, and by providing a high-resolution readout for functional perturbation experiments in human organoids.

To realize the full potential of human organoids, key challenges need to be addressed (Fig. 1c). Most immediately, we need better characterization and validation of organoids as faithful models of human biology. This will require assays for informative high-throughput profiling as well as the definition of quality standards for cell composition, cellular differentiation, cell states and responses to stimuli. A future catalog of well-characterized human organoids should include extensive replication, to quantify technical and biological sources of variation. Moreover, inclusion of genetically diverse sample donors will help to assess interindividual variation in the human population.

Current organoid protocols, while useful for many applications, have relevant technical and conceptual limitations. For example, organoids may not faithfully represent the diversity of cell types in primary tissue (including non-parenchymal cells such as immune cells and stroma), and they are limited in their ability to account for the effects that environmental exposures and organismal aging have on human organs in vivo. It will be important to develop robust protocols that yield organoids with adequate tissue organization, differentiated cells, vascularization, immune cell infiltration and, for some organs (for example, skin and intestine), even a microbiome. Moreover, we are still learning how to use organoids most effectively for discovering biology (for example, through genetic and pharmacological perturbations) and how to exploit them for drug development and personalized medicine.

Single-cell sequencing and spatial profiling have a key role in addressing these issues (Fig. 1d). Comprehensive molecular maps of organoids and organoid development can reveal cell states and transcription regulatory programs in unprecedented detail, and comparisons to corresponding human tissues in vivo provide powerful new ways of evaluating organoids. Single-cell epigenome and transcriptome profiling yields a quantitative, high-dimensional assessment of cell composition and cell states within organoids. Spatial profiling assays characterize tissue organization and three-dimensional architecture. These methods also enhance organoid quality control (for example, identifying outliers, missing cell types or aberrant gene regulation), and they can provide reference atlases for disease-centric studies. Furthermore, comparative molecular profiling of organoids and matched ex vivo tissue samples can guide the development of new and improved organoid protocols, for example, by identifying missing cell populations or bottlenecks of cellular differentiation in organoids. Finally, single-cell technologies provide a powerful and scalable readout for functional experiments and for genetic or pharmacological perturbations that are tested in human organoids.

To demonstrate the feasibility and utility of combining human organoids with single-cell technology, we have launched an Organoid Cell Atlas pilot project, as a ‘Biological Network’ within the Human Cell Atlas (HCA)^4–6^, focusing on the single-cell characterization of organoids and complex in vitro systems (https://www.humancellatlas.org/coordinators). The Organoid Cell Atlas will facilitate the production, quality control, dissemination and utilization of single-cell and spatial genomics data for human organoids, and it will link such datasets to the comprehensive profiles of primary tissues that are being generated within the HCA. A first step toward establishing the Organoid Cell Atlas has recently been funded by the European Union Horizon 2020 call for “Pilot actions to build the foundations of a human cell atlas” with the ‘HCA|Organoid’ project (https://hca-organoid.eu).

## The Organoid Cell Atlas within the HCA

The Organoid Cell Atlas will be a cornerstone of the HCA. It is central to the HCA’s mission of creating comprehensive reference maps of all human cells as a basis for understanding human health and for diagnosing, monitoring and treating disease. Like the Human Genome Project^7^, the HCA is expected to have substantial biomedical impact, most notably in areas such as precision medicine and regenerative biology. Human organoids will be of great importance in the HCA^6^, as they complement the profiling of primary tissue samples with a readily perturbed model for functional studies while also benefitting from the profiling of human tissues to which they can be compared.

Some of the first applications of single-cell profiling in organoids included single-cell RNA-seq analysis of the cellular composition of mouse intestinal organoids^8^ and human brain organoids^9^. Other pioneering studies uncovered complex cellular networks^10^ and used single-cell profiles to improve organoid derivation protocols^11^. These initial reports identified striking parallels among organoid development, human primary tissue and in vivo organogenesis, which reinforces the potential of organoids for dissecting human biology in vitro.

To exploit synergies between single-cell profiling and organoid technologies, the Organoid Cell Atlas has been initiated in close coordination with the HCA leadership, following the example of other specialized atlases such as the Pediatric Cell Atlas^12^ and the Human Tumor Cell Atlas Network^13^. The Organoid Cell Atlas is an open, collaborative network that pursues four complementary directions: first, encourage and standardize single-cell profiling of human organoids; second, facilitate access to single-cell organoid data (and informative metadata) via HCA data infrastructure; third, establish computational methods and tools for connecting organoid profiles with primary tissue data; and fourth, put organoids into their biological context using HCA profiles of their in vivo counterparts. The envisioned data integration will be driven by an interactive, openly accessible, web-based computational platform called the Organoid Cell Atlas Portal, which will build on and extend the existing HCA data infrastructure.

## An initial version of the Organoid Cell Atlas

**Fig. 2:**
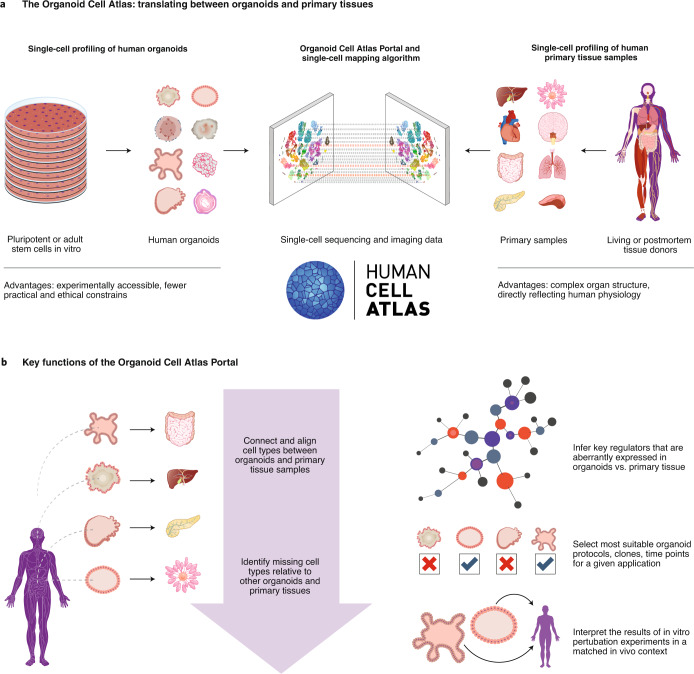
Connecting single cells in human organoids and in primary tissue samples via the Organoid Cell Atlas. **a**, Single-cell profiling of human organoids (left) and of human primary tissue samples (right) provides complementary information. Data integration between single-cell profiles from organoids and primary tissues makes it possible to investigate the same cell type in both contexts, allowing each approach to play to its strengths. **b**, The Organoid Cell Atlas Portal will implement key features for analyzing and interpreting single-cell data from human organoids in the biological context provided by HCA profiles of their in vivo counterparts.

The European Union Horizon 2020 (EU H2020) HCA|Organoid project enables us to establish a first version of the Organoid Cell Atlas over the coming years, which may act as a nucleus for a broader, collaborative, global initiative. In this pilot project, we are generating single-cell transcriptome profiles, epigenome maps and detailed imaging data in a selection of human organoids. For two organs, colon and brain, we will derive and characterize organoids from 100 whole-genome-sequenced individuals each, to capture normal population variation and to establish a reference for disease-centric studies (Fig. 2a,b). User-friendly access to the single-cell data for organoids and comparisons between organoid profiles and single-cell data for human primary tissues will be available on the Organoid Cell Atlas Portal, which is being developed in parallel with the data generation.

We selected colon and brain organoids as the two focus areas of the HCA|Organoid project for three reasons: first, colon and brain were among the first organs for which organoids were demonstrated, so relatively mature protocols are now available; second, colon organoids are derived from adult stem cells in primary ex vivo samples while brain organoids are derived from pluripotent cells, thus spanning the two main sources of organoid derivation; third, colon and brain organoids have already been used for disease-centric studies, and single-cell characterization of these organoids for a large number of individuals will facilitate biomedical applications. Beyond the initial focus on colon and brain organoids, the HCA|Organoid project is designed in such a way that most of the data infrastructure is generic and applicable to other types of human organoids. We actively seek collaboration with other projects that pursue systematic single-cell profiling in other types of human organoids to explore the possibility of interconnection or integration with the Organoid Cell Atlas.

The Organoid Cell Atlas Portal is a central aim of the EU H2020 HCA|Organoid project. It will provide computational infrastructure and a web-based front end that make single-cell data for human organoids easy to access and analyze. This effort will build upon the existing Data Coordination Platform infrastructure of the HCA (https://data.humancellatlas.org) for data submission, processing, annotation and retrieval. Key features that are specific to organoids will include the interactive exploration of human organoid data, data-driven selection of organoids for functional experiments, and comparison of disease-specific organoids against reference collections of normal organoids.

The Organoid Cell Atlas Portal will also provide interactive mappings between single-cell profiles of human organoids and the corresponding primary tissues available within the HCA, using algorithms that enable cell–cell alignments between these datasets. This functionality will facilitate and encourage the use of organoids as a model for detailed biological experiments, including the identification of target genes for mechanistic research and drug development. The mapping and data integration will also allow exploration of normal variation between individuals (for example, due to common genetic differences) in an interactive manner, leveraging organoids as a model for the corresponding variation in primary tissues. Finally, the cell–cell alignments will facilitate the analysis and interpretation of perturbations in human organoids in the context of the corresponding primary tissues.

We will pursue several complementary strategies to ensure that the data in the Organoid Cell Atlas will be of the highest possible quality and reproducibility. First, we will invest in standardization and validation of experimental workflows for organoid derivation — for example, by comparing alternative protocols and by assessing the relative effect of technical and biological factors on the single-cell profiles of colon and brain organoids. Second, we will contribute to HCA efforts to establish community standards and software infrastructure for data processing and data annotation — for example, by ensuring compatibility with the specific metadata structure for organoids. Third, we will develop and validate computational methods for the flexible alignment and comparison of cells between organoids and corresponding primary tissue. Finally, we will implement interactive visualization tools that enable user-friendly quality control and exploratory analysis of single-cell organoid datasets contributed to the Organoid Cell Atlas.

To maximize the utility and impact of the EU H2020 HCA|Organoid project for the broader scientific community, single-cell profiles will be made public as rapidly as possible, in concordance with the HCA’s strong commitment to data sharing, local ethical regulations and the European data protection law (GDPR). Newly established organoids will be provided as a ‘living biobank’ via Hubrecht Organoid Technology^14^ (colon) or as a set of precise protocols for derivation from biobanked induced pluripotent stem cell lines (brain). Furthermore, we will evaluate and explore the practical utility of the Organoid Cell Atlas in a series of disease-centric pilot studies, pursuing CRISPR screening with single-cell transcriptome readout (CROP-seq)^15^ and disease modeling of genetic epilepsy using brain organoids selected from the Organoid Cell Atlas. Finally, we are committed to developing the Organoid Cell Atlas Portal into a public, sustainable and widely used infrastructure for finding, accessing, analyzing and interpreting single-cell data from human organoids.

## Future directions

With recent technological advances, human beings are becoming the leading ‘model organism’ for the discovery of important new biology and the development of therapeutic strategies. The HCA is catalyzing this paradigm shift by establishing reference maps of all human cells, for the first time providing a detailed molecular picture of the human body. In vitro models such as organoids will complement these efforts by enabling researchers to functionally dissect and systematically perturb human biological systems. In the context of the HCA, the EU H2020 HCA|Organoid project is committed to establishing a practically useful and readily extensible initial version of the Organoid Cell Atlas within two years, while extensive further work will be needed to maximize the impact of the Organoid Cell Atlas for basic biology and biomedical applications. Beyond the initial focus on colon and brain, the Organoid Cell Atlas will incorporate datasets from other organoids and organ systems through the HCA data infrastructure and in collaboration with the other HCA Biological Networks.

The mission of the Organoid Cell Atlas is to advance biomedical discovery and the development of regenerative therapies by supporting and accelerating disease-centric research in areas such as rare genetic diseases, complex multifactorial diseases and precision oncology. This overarching goal can be achieved by creating an open and inclusive research environment that facilitates collaboration among a broad range of interested researchers, bridging communities and integrating expertise in organoids and single-cell technology.
